# Personal

**Published:** 1863-03

**Authors:** 


### PERSONAL.

Our friend, E. T. Lovering, of Toland's Dental Depot notoriety, is, we are sorry to learn, about to leave that establishment, and enter upon a more migratory calling. He has been engaged by the American Hard Rubber Company as a traveling agent amongst the dental fraternity of the East, and we are confident from several years acquaintance with him, both as a gentleman and a business man, that he is eminently qualified for such a work. He is well acquainted with the principles of dentistry, familiar with all the materials used by dentists, and well knows their wants. He is well known to the profession throughout the west. He has the ability to make himself acceptable every where. T.

